# Circular RNA hsa_circ_0000073 Enhances Osteosarcoma Cells Malignant Behavior by Sponging miR-1252-5p and Modulating CCNE2 and MDM2

**DOI:** 10.3389/fcell.2021.714601

**Published:** 2021-09-09

**Authors:** Zhijing Ren, Qinqin Yang, Jiajia Guo, Haifeng Huang, Bo Li, Zhen Yang, Xiaobin Tian

**Affiliations:** ^1^Medical College of Guizhou University, Guiyang, China; ^2^Department of Clinical Laboratory, Guizhou Provincial People’s Hospital, Guiyang, China; ^3^Department of Orthopedics, Guizhou Provincial People’s Hospital, Guiyang, China; ^4^Department of Orthopedics, Affiliated Hospital of Guizhou Medical University, Guiyang, China

**Keywords:** hsa_circ_0000073, osteosarcoma, cell cycle, proliferation, miR-1252-5p, CCNE2, MDM2

## Abstract

**Objective:** An increasing number of studies have demonstrated that circular RNAs (circRNAs) are involved in tumor progression. However, the role of hsa_circ_0000073 in osteosarcoma (OS) is still not fully elucidated.

**Methods:** Quantitative reverse transcription-polymerase chain reaction or Western blot was used to detect the gene expression. GeneChip analysis, bioinformatics, luciferase reporter, and RNA immunoprecipitation assays were adopted to predict and verify the relationships between genes. Counting Kit-8 Assay, clone formation assay, wound-healing assay, transwell assays, cell cycle assays, and *in vivo* tumorigenesis were used to evaluate cell function.

**Results:** hsa_circ_0000073 was highly expressed in OS cell lines and could promote OS progression, including proliferation, migration, invasion, and cell cycle *in vitro* as well as tumorigenesis *in vivo*. Mechanically, hsa_circ_0000073 could readily downregulate the expression of CCNE2 and MDM2 through miR-1252-5p. Rescue experiments validated miR-1252-5p mimics, or CCNE2/MDM2 short hairpin RNA could reverse the hsa_circ_0000073 overexpressing-induced impairment of malignant tumor behavior.

**Conclusion:** hsa_circ_0000073 functions as a tumor promoter in OS to increase malignant tumor behavior through sponging miR-1252-5p and regulating CCNE2 and MDM2 expression, which could be a novel target for OS therapy.

## Introduction

Osteosarcoma (OS), the most frequent bone tumor from malignant mesenchymal cells, is the leading cause of cancer mortality in children and teenagers. Unfortunately, although advanced surgery combined with chemotherapy has been practiced in clinical, the patients with OS have been shown to only approximately 65–70% in 5-year survival rate, and many patients suffer from potential distant metastasis (Zhou et al., 2016; Harrison et al., 2018).

It is widely accepted that patients with OS may benefit from the novel and efficacious treatment methods established, such as molecule-targeted therapies. However, little progress has been made in recent decades. Therefore, there is a pressing need to profoundly investigate the molecular mechanism underlying OS progress, especially complex gene regulation axes, which could help us develop robust interventions and therapies (Bishop et al., 2016; Otoukesh et al., 2018).

Circular RNAs (circRNAs) are a subclass of endogenous non-coding RNAs with no polyadenylated tail, which have a closed circular structure joined by the 3′ and 5′ ends (Kristensen et al., 2019). Increasing evidence has exhibited that multiple circRNAs have been involved in the generation and development of cancers, such as gene expression, migration, invasion, proliferation, cell cycle, among others (Arnaiz et al., 2019; Chen et al., 2020; Li R. et al., 2020; Wu P. et al., 2020). Currently, as many micro RNA (miRNA)-binding sites on circRNAs have been found, the most well-known function of circRNAs is to serve as miRNA sponges and subsequently regulate target gene expression (Lu et al., 2019; Wang H. Y. et al., 2020). In OS, extensive research has demonstrated that circRNAs could participate in the pathophysiological processes by exhibiting competitive binding to miRNAs, such as circ-XPR1, circTADA2A, hsa_circ_0005909, and hsa_circ_0000658 (Wu et al., 2019; Jiang and Chen, 2021; Mao et al., 2021; Zhang et al., 2021). However, studies of circRNAs in OS are just starting, and the underlying molecular mechanism of hsa_circ_0000073 is not fully understood. Here, we aimed to explore the function of hsa_circ_0000073 in proliferation, migration, invasion, and cell cycle of OS cells and unveiled a network among hsa_circ_0000073/miR-1252-5p/CCNE2 and MDM2, providing a potential target for OS therapy.

## Materials and Methods

### Cell Culture

A normal human osteoblast cell line (hFOB1.19), three OS cell lines (MG63, U-2, and Saos2), were obtained from the Chinese Academy of Sciences (Shanghai, China) and cultured under standard conditions (temperature: 37°C; CO: 2.5%). For a complete medium, 100 U/ml penicillin G, streptomycins, and 10% fetal bovine serum were mixed in the Dulbecco’s modified Eagle’s medium (Gibco, United States).

### Transfection of Cells

For transfection experiments, the empty vector (pcDNA3.1), overexpressing plasmids (hsa_circ_0000073), short hairpin RNAs (shRNAs) (sh-NC, sh-circ_0000073, sh-MDM2, and sh-CCNE2), miRNA mimics, and sponge (mimics-NC, miR-1252-5p mimics, sponge-NC, and miR-1252-5p sponge) were all designed and synthesized by General Biosystems (Anhui, China). Lipofectamine 3000 (Invitrogen, United States) was chosen for cell transfection. The shRNAs used are shown in Supplementary Table 1.

### RNase R Treatment and Quantitative Reverse Transcription-Polymerase Chain Reaction

TRIzol (Takara, Japan) was taken for total RNA extraction. At 37°C, 2,000 ng of total RNA was incubated with or without RNase R (Epicenter Technologies, United States) for 15 min. The reverse transcription kit (Takara, Japan) and an SYBR Green PCR kit (Takara, Japan) were used for quantitative reverse transcription-polymerase chain reaction (qRT-PCR). The expression levels were normalized with glyceraldehyde 3-phosphate dehydrogenase or U6 and calculated with the 2^–ΔΔCt^. The primers used are displayed in Supplementary Table 2.

### Counting Kit-8 Assay

Counting Kit-8 (CCK-8) reagent (Solarbio, China) was chosen to test cell proliferation. The transfected cells were cultured in 96-well plates. At 0, 24, 48, and 72 h, 10 μl of CCK-8 reagent was added to each well. After 2 h of incubation at 37°C, a microplate reader was taken to measure the optical density value at 450 nm.

### Clone Formation Assay

Transfected cells were plated in 12-well plates for 1-week culture before being fixed in 4% paraformaldehyde. Crystal violet solution (0.1%, Solarbio, China) was used to stain. The images were captured for counting.

### Wound-Healing Assay

Cells in the different groups were cultured in 12-well plates for 24 h. A 10-μl pipette tip was taken to scratch the cell surface. At 0 and 48 h after injury, the images were captured by a microscope. A relative migration rate was analyzed by measuring the migratory distance normalized to the 0-h control.

### Transwell Assay

A transwell chamber coating matrigel on the upper side was taken to examine the cell invasion. The transfected cells with 200-μl serum-free media were added into the transwell chambers, whereas the outer chambers were packed with the complete medium. After 48 h of culture, the bottom surface cells were fixed with 4% paraformaldehyde and stained with 0.1% crystal violet (Solarbio, China). The images were captured for counting.

### Cell Cycle Assay

The flow cytometer and a cell cycle analysis kit (Meilunbio, China) were used to detect the cell cycle stages. The cells were immobilized with 75% alcohol at −20°C for 24 h and added 500-μl propidium iodide solution [buffer:propidium iodide (20×):RNase A (50×) = 100:5:2] and incubated 30 min for the test.

### Gene Expression Profiling Analysis and Bioinformatics Analysis

Cells transfected with sh-NC or sh-circ_0000073 were subjected to total RNA isolation using TRIzol reagent (Takara, Japan). Then, a GeneChip WT Pico Reagent Kit (Affymetrix, United States) was taken to analyze the differentially expressed messenger RNAs with the conditions: fold change > 1.5 and *P* < 0.05. Gene Ontology (GO) (Yu et al., 2012) and Kyoto Encyclopedia of Genes and Genomes (KEGG) (Kanehisa et al., 2021) were taken to investigate the potential biological functions of hsa_circ_0000073 with the conditions: p.adj < 0.05 and *q*-value < 0.2. Protein–protein interaction (PPI) network and competing for endogenous RNA (ceRNA) network were created by STRING 11.0 (Szklarczyk et al., 2019) and Cytoscape 3.7.2 (Shannon et al., 2003).

### Western Blot

Radioimmunoprecipitation assay buffer and BCA Protein assay kit (Beyotime, China) were used for protein extraction and protein concentration evaluation. Proteins were separated with a sodium dodecyl sulfate–polyacrylamide gel electrophoresis gel. The polyvinylidene fluoride membrane containing proteins was blocked with 5% milk. Then, specific primary antibodies (MDM2, Abcam; CCNE2, Abcam; and glyceraldehyde 3-phosphate dehydrogenase, Abcam) were applied to the membrane at 4°C overnight. After incubating with secondary antibodies, an ECL Western Blotting Substrate (Solarbio, China) was used to detect the protein blots.

### RNA Immunoprecipitation

An EZ-Magna RNA Immunoprecipitation (RIP) Kit (Millipore, United States) was taken to AGO2-RIP experiments. The HEK-293 cells were lysed and incubated with human anti-Ago2 or mouse IgG-coated beads (Millipore, United States). qRT-PCR was used to analyze the immunoprecipitated RNAs.

### Luciferase Reporter Assay

According to the target gene sites identified *via* Circbank (Liu et al., 2019), TargetScan (Agarwal et al., 2015), or ENCORI (Li et al., 2014), the wild-type or mutant sequence was plug into the vector (Tongyong, China) and transfected to cells. A double luciferase detection system was measured to the activity of the luciferase reporter gene.

### Tumor Formation *in vivo*

BALB/C-nu mice (Sipeifu, China) were used to study tumor formation ability, in which 2 × 10^7^ stably transfected MG63 cells were injected in the flank for 5 weeks. The tumor volume was monitored every week and calculated using the formula: length × width^2^/2 (cubic millimeter). On week 5, mice were killed. The dissected tumors were weighed and collected. Formalin-fixed paraffin sections (4–6 μm) of tumor tissues were carried out for immunohistochemistry assay. The primary antibody (MDM2, Abcam; CCNE2, Abcam) and EnVision Detection System (DAKO, United States) were used according to the manufacturer’s protocol. A microscope captured images. The Animal Ethics Committee of Guizhou Provincial People’s Hospital approved this work.

### Statistical Analysis

The data were represented as means ± SD and the Student’s *t*-test, or one-factor analysis was taken to analyze the group comparison. Through SPSS 22.0 (IBM, United States) analysis, statistical significance was recording as *P* < 0.05.

## Results

### hsa_circ_0000073 Was Upregulated in Osteosarcoma Cells

**FIGURE 1:**
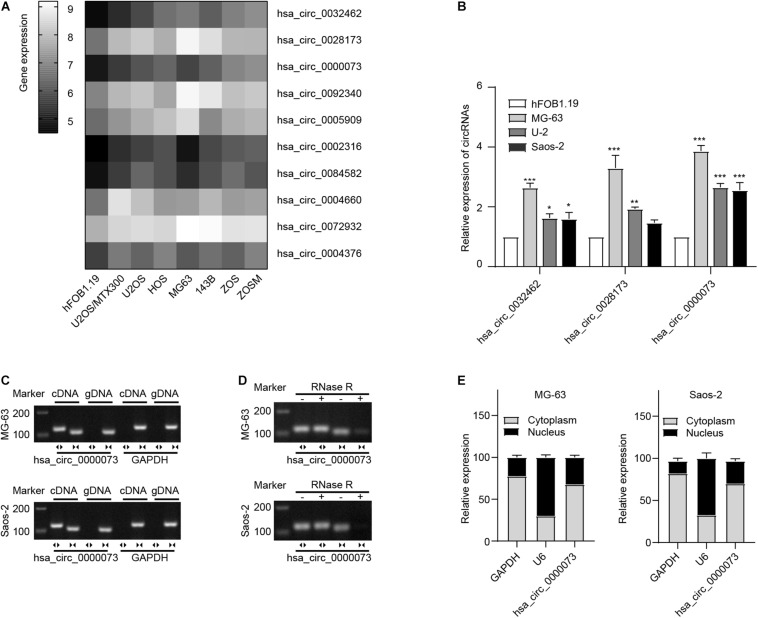
hsa_circ_0000073 was upregulated in OS cells. **(A)** Clustered heatmap displayed most upregulated circRNAs in OS cell lines from GSE96964. **(B)** qRT-PCR demonstrated a higher expression level of hsa_circ_0000073 in OS cells. **(C,D)** AGE indicated existence and confirmed circular structure of hsa_circ_0000073. **(E)** qRT-PCR showed hsa_circ_0000073 was predominantly localized in cytoplasm. Data were presented as mean ± SD. *P < 0.05, **P < 0.005, ***P < 0.001.

We analyzed the most upregulated circRNAs in OS cell lines from GSE96964 (Figure 1A). The hsa_circ_0032462, hsa_circ_0028173, and hsa_circ_0000073 were selected to further investigate by qRT-PCR. Comparing with hFOB1.19, the normal human osteoblast cell, the highest expression of hsa_circ_0000073 was observed in human OS cells, including MG63, U2OS, and Saos2 (Figure 1B). Then, hsa_circ_0000073 was chosen to be the main subject. Subsequently, we confirmed that hsa_circ_0000073 could only be detected from complementary DNA with divergent primers (Figure 1C) and undigested by RNase R, which showed strong stability due to the closed structure (Figure 1D). Moreover, the data of qRT-PCR proved that hsa_circ_0000073 was enriched in the cytoplasm (Figure 1E).

### hsa_circ_0000073 Promoted Osteosarcoma Progression *in vitro*

**FIGURE 2:**
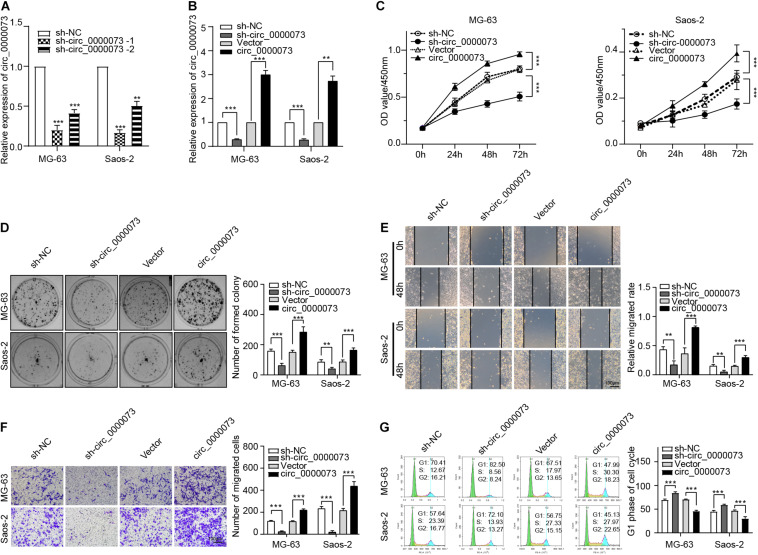
hsa_circ_0000073 enhanced malignant behavior *in vitro*. **(A)** qRT-PCR qualified silenced efficiencies of hsa_circ_0000073. **(B)** qRT-PCR confirmed knockdown and overexpression of hsa_circ_0000073 by shRNA and overexpressing plasmid. **(C,D)** CCK-8 and clone formation assays showed hsa_circ_0000073 stimulated cell proliferation in OS cells. **(E,F)** Wound-healing and transwell assays verified hsa_circ_0000073 enhanced migration and invasion ability in OS cells with regulation, magnification, ×200. **(G)** Flow cytometry analysis revealed that hsa_circ_0000073-silenced induced S phase arrested in OS cells, whereas hsa_circ_0000073-overexpressed reductions. Data were presented as mean ± SD. **P < 0.005, ***P < 0.001.

Then, we designed two shRNAs that targeted the specific junction sites of hsa_circ_0000073. By qRT-PCR, we found that the sh-hsa_circ_0000073-1 had a better knockdown efficiency (Figure 2A). Thus, we chose the sh-hsa_circ_0000073-1 for further study. By contrast, we also designed an overexpressing plasmid of hsa_circ_0000073, which was verified that it could significantly upregulate the expression of hsa_circ_0000073 in OS cells (Figure 2B). Functionally, our results of the CCK-8 and colony formation assays confirmed that the hsa_circ_0000073 shRNA prominently weakened the cell proliferative ability, and the overexpression group was the opposite (Figures 2C,D). Moreover, the wound-healing and transwell assays also showed that the migrative and invasive abilities were similar to CCK-8 and colony formation assays (Figures 2E,F). Next, flow cytometry found that the S phase was higher by hsa_circ_0000073-silenced, whereas it was lower by overexpressing hsa_circ_0000073 (Figure 2G). In a word, hsa_circ_0000073 played an oncogenic role in OS progression.

### Bioinformatics Predicted the Competing for Endogenous RNA Network of hsa_circ_0000073

**FIGURE 3:**
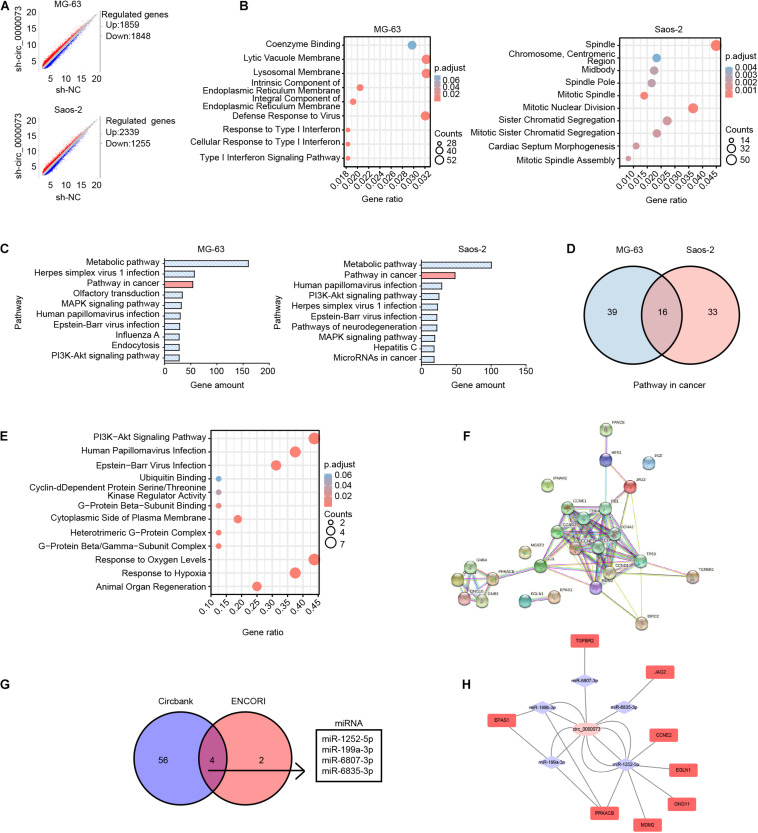
Bioinformatics analysis of DEGs after hsa_circ_0000073 silencing. **(A)** Scatter plot of all DEGs between sh-NC group and sh- circ_0000073. **(B)** GO analyses showed enrichment of down-regulated DEGs. **(C)** KEGG pathways analysis of top 10 pathways. **(D)** Venn analysis revealed amount genes in “pathways in cancer” of two cell lines. **(E)** GO and KEGG analyses displayed enrichment of co-expressed DEGs. **(F)** PPI network analysis showed interaction of co-expressed DEGs as well as relational genes. **(G)** Databases predicted four miRNAs shared with hsa_circ_0000073. **(H)** ceRNA network displayed relationship among hsa_circ_0000073, miRNA, and target genes collected by co-expressed DEGs in “pathway in cancer.”

To explore the complex underlying mechanisms, we first analyzed the gene expression profiling after transfected sh-hsa_circ_0000073 in MG-63 and Saos-2 cells. After hsa_circ_0000073 was silenced, the scatter plot showed there were 1,859 upregulated and 1,848 downregulated genes in MG-63 cells, as well as 2,339 upregulated and 1,255 downregulated genes in Saos-2 cells (Figure 3A). Subsequently, GO analyses showed that downregulated differentially expressed genes (DEGs) were enriched in a diverse cellular component, molecular function, or biological process (Figure 3B). Further KEGG pathways analysis indicated that “pathways in cancer” were one of the major pathways in both two cell lines (Figure 3C). Moreover, the Venn analysis revealed 16 genes in “pathways in cancer” that were co-downregulated in the two cell lines (Figure 3D). The details are displayed in Supplementary Table 3. By GO and KEGG analyses, the co-expressed DEGs were involved in cyclin-dependent protein, ubiquitin-binding, GTPase complex, and PI3K-Akt signaling pathway, which might imply to connect with cell growth and differentiation (Figure 3E). PPI network analysis showed the interaction of the 16 co-expressed DEGs and their relational genes (Figure 3F); the details of PPIs are shown in Supplementary Table 4.

Considering circRNAs are important in competing endogenous RNA networks, we searched the databases and found four miRNAs with several potential binding sites of hsa_circ_0000073 in circBank and ENCORI (Figure 3G). The ceRNA network analysis displayed the relationship among hsa_circ_0000073, miRNA, and target genes collected by co-expressed DEGs in “pathway in cancer” (Figure 3H). To sum up, based on bioinformatics, we made a potential ceRNA network of hsa_circ_0000073 that pointed out the direction for further research.

### miR-1252-5p Combined With hsa_circ_0000073 and Suppressed Osteosarcoma Progression

**FIGURE 4:**
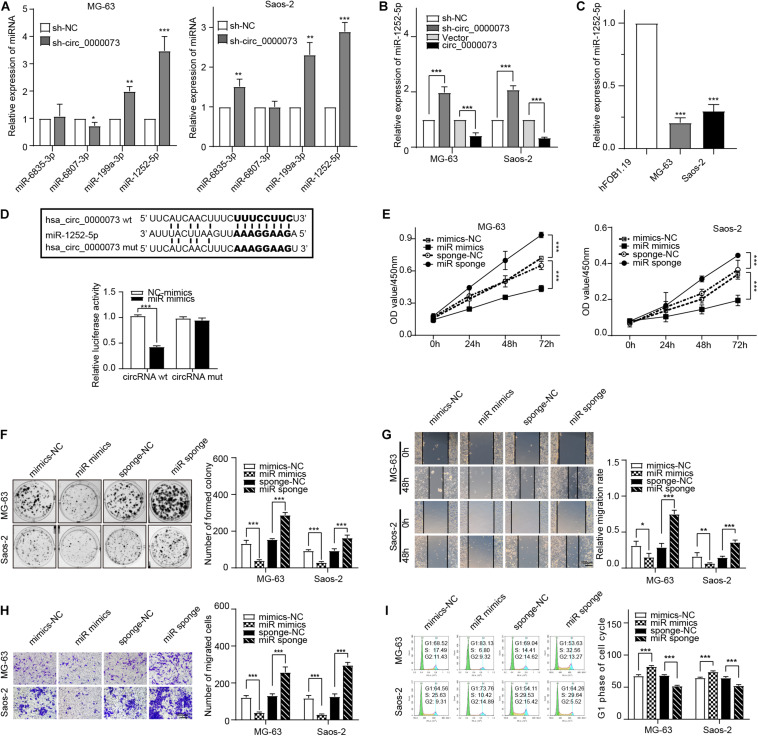
hsa_circ_0000073 targets miR-1252-5p to promote OS progress. **(A)** qRT-PCR examined expression of four candidate miRNAs after hsa_circ_0000073 silencing. **(B)** qRT-PCR measured expression of miR-1252-5p in MG-63 and Saos-2 cells after overexpression or knockdown of hsa_circ_0000073. **(C)** qRT-PCR confirmed lower expression level of miR-1252-5p in OS cells. **(D)** Luciferase reporter assay confirmed relationship between hsa_circ_0000073 and miR-1252-5p in HEK293 cells. **(E,F)** CCK-8 and clone formation assays showed miR-1252-5p inhibited cell proliferation in OS cells. **(G,H)** Wound-healing and transwell assays revealed miR-1252-5p restrained migration and invasion ability in OS cells with regulation, magnification, ×200. **(I)** Flow cytometry analysis indicated upregulation of miR-1252-5p induced S phase arrested in OS cells whereas downregulation of reductions. Data were presented as mean ± SD. *P < 0.05, **P < 0.005, ***P < 0.001.

Subsequently, we checked the expression of the four miRNAs after the knockdown of hsa_circ_0000073. The results of qRT-PCR uncovered that miR-1252-5p was significantly upregulated by hsa_circ_0000073 repression and was lowly expressed in OS cells (Figures 4A–C). Moreover, the binding between miR-1252-5p and hsa_circ_0000073 was further verified by the luciferase assay (Figure 4D). Thus, we chose miR-1252-5p for further study and designed the effective mimics and sponge of it (Supplementary Figure 1). Functionally, we further investigated the role of miR-1252-5p in OS progression, which found that miR-1252-5p could inhibit the carcinoma progression in proliferation, migration, invasion, and cell cycle (Figures 4E–I). Taken together, our study found and supported that miR-1252-5p could combine with hsa_circ_0000073 and suppresses the malignant behavior of OS cells.

### hsa_circ_0000073 Sponging miR-1252-5p and Upregulated CCNE2 and MDM2 Expression

**FIGURE 5:**
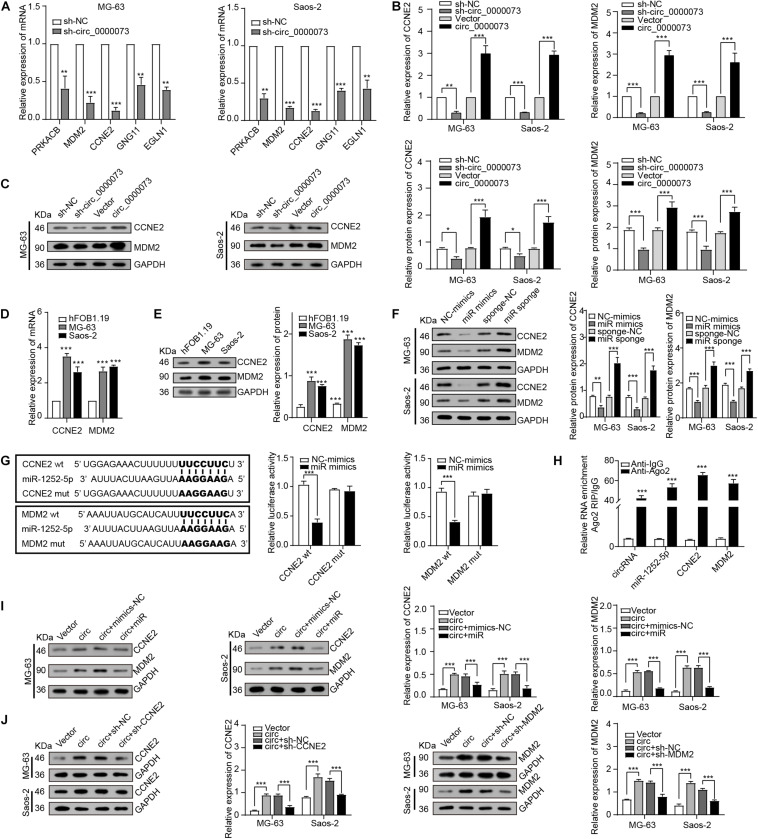
hsa_circ_0000073 regulated expression of CCNE2 and MDM2 through miR-1252-5p. **(A)** qRT-PCR determined expression of five candidate genes after hsa_circ_0000073 silencing. **(B,C)** qRT-PCR and WB assays assessed expression of CCNE2 and MDM2 in OS cells with hsa_circ_0000073 regulation. **(D,E)** qRT-PCR and WB assays confirmed expression level of CCNE2 and MDM2 was higher in OS cells. **(F)** WB verified impacts of miR-1252-5p on CCNE2 and MDM2 expression in OS cells. **(G,H)** Luciferase reporter assay and RIP assays confirmed relationship among hsa_circ_0000073, miR-1252-5p, CCNE2, and MDM2 in HEK293 cells. **(I,J)** WB revealed rescue ability of miR-1252-5p, CCNE2, and MDM2 on overexpressed-hsa_circ_0000073 in MG-63 and Saos-2 cells. Data were presented as mean ± SD. *P < 0.05, **P < 0.005, and ***P < 0.001.

Next, based on the ceRNA network predicted by bioinformatics, the five genes related to miR-1252-5p were selected for further investigation, which found CCNE2 and MDM2 were the most dramatically downregulated genes by hsa_circ_0000073 silencing (Figure 5A). The further results of qRT-PCR and Western blot (WB) assay confirmed that hsa_circ_0000073 silencing observably downregulated the expression of CCNE2 and MDM2, whereas its overexpressing plasmid was the opposite (Figures 5B,C). Besides, we revealed that CCNE2 and MDM2 were dramatically increased in the OS cells and could be regulated by miR-1252-5p (Figures 5D–F). Furthermore, luciferase assay and RIP assays also confirmed the binding among hsa_circ_0000073, miR-1252-5p, CCNE2, or MDM2 (Figures 5G,H). Thus, we chose CCNE2 and MDM2 for further study and designed the effective shRNAs (Supplementary Figure 2). Moreover, the WB data validated that hsa_circ_0000073-overexpressed could upregulate the expression of CCNE2 and MDM2, and this upregulation could be attenuated by miR-1252-5p-overexpressed, CCNE2-silenced, or MDM2-silenced (Figures 5I,J). Collectively, our findings suggested that hsa_circ_0000073 could affect the expression of CCNE2 and MDM2 by regulating miR-1252-5p.

### hsa_circ_0000073 Enhanced the Malignant Behavior of Osteosarcoma Cells by Targeting miR-1252-5p/CCNE2 and MDM2 Axis

**FIGURE 6:**
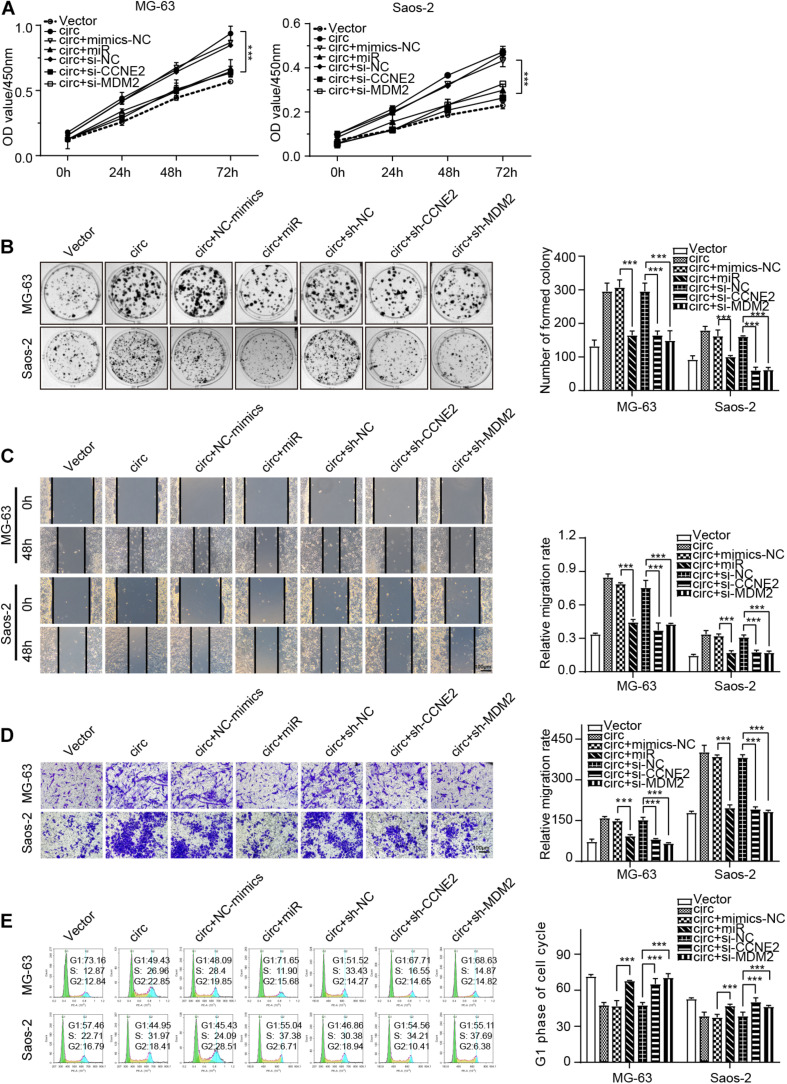
hsa_circ_0000073 enhanced malignant behavior of OS cells by targeting miR-1252-5p and regulating CCNE2 and MDM2. **(A,B)** CCK-8 and colony formation assays demonstrated that overexpressed-miR-1252-5p, silenced-CCNE2, or MDM2 restored growth promotion induced by hsa_circ_0000073 overexpression. **(C,D)** Wound-healing and transwell assays evaluated cell migration and invasion in indicated groups, magnification, ×200. **(E)** Flow cytometry assay determined cell cycle under diverse conditions. Data were presented as mean ± SD ***P < 0.001.

Functionally, rescue experiments were performed to explore whether hsa_circ_0000073 could participate in the progress of OS by sponging miR-1252-5p and regulating CCNE2 or MDM2 expression. The data of CCK-8, colony formation, transwell, wound-healing, and cell cycle assays verified that miR-1252-5p mimics, sh-CCNE2, or sh-MDM2 could reverse the malignant behavior caused by hsa_circ_0000073 overexpression in OS cells (Figures 6A–E). Overall, our results proved that hsa_circ_0000073 regulated CCNE2 or MDM2 through miR-1252-5p.

### hsa_circ_0000073 Promotes the Tumorigenesis Through Sponging miR-1252-5p and Modulating CCNE2 and MDM2 Expression *in vivo*

**FIGURE 7:**
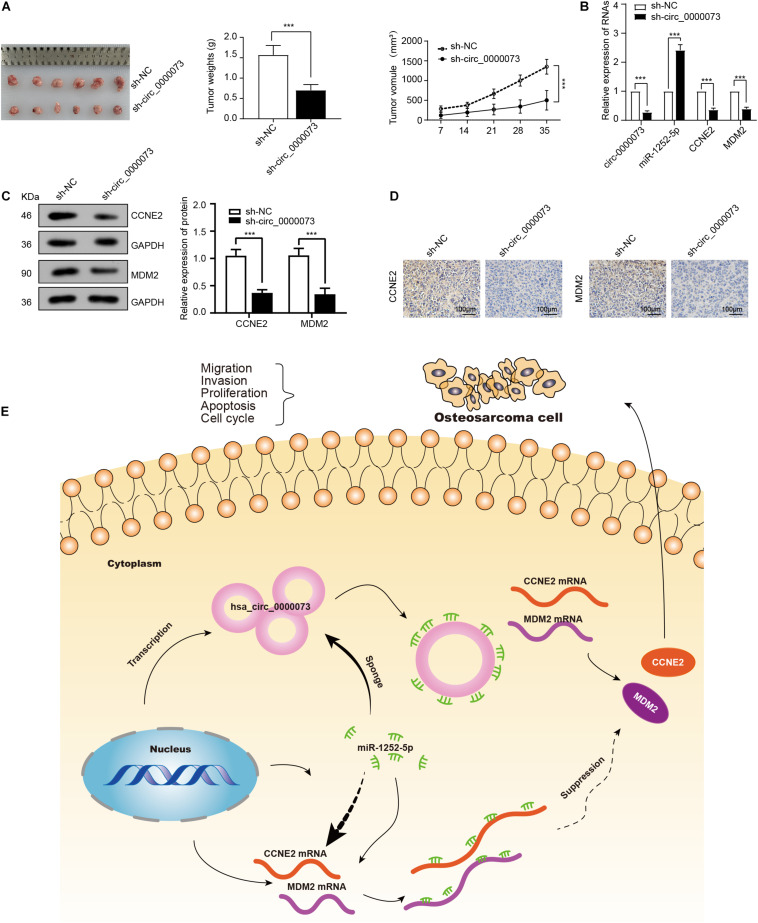
Knockdown of hsa_circ_0000073 represses tumor growth through sponging miR-1252-5p and regulating CCNE2, and MDM2 *in vivo*. **(A)** Images, weight and growth curve of tumors after transfection with sh- hsa_circ_0000073 or sh-NC. **(B)** qRT-PCR determined expression of hsa_circ_0000073, miR-1252-5p, CCNE2, and MDM2 in tumors collected earlier. **(C,D)** WB and immunohistochemistry evaluated expression of CCNE2 and MDM2 when hsa_circ_0000073 was silenced, magnification, ×200. **(E)** Schematic illustration of final regulatory circuit among hsa_circ_0000073, miR-1252-5p, CCNE2, and MDM2. Data were presented as mean ± SD ***P < 0.001.

A xenograft tumor model showed that the tumors derived from the cells transfected sh-hsa_circ_0000073 weighed less and grew more slowly than the control group (Figure 7A). Next, qRT-PCR confirmed the expression of miR-1252-5p was upregulated by hsa_circ_0000073 knockdown (Figure 7B). Meanwhile, the results of WB and immunohistochemistry showed that hsa_circ_0000073 silencing markedly reduced the expression of CCNE2 or MDM2 in tumor tissues (Figures 7C,D). To summarize, hsa_circ_0000073 enhances osteosarcoma cells’ malignant behavior by sponging miR-1252-5p and modulating CCNE2 and MDM2 (Figure 7E).

## Discussion

Circular RNAs are an interesting class of RNA that do not have free ends. This special structure gives them excellent stability. An *in vitro* study of 60 circRNAs confirmed that most circRNAs have longer half-lives than linear RNAs (Enuka et al., 2016). Their high stability, abundance, and many other characteristics make them become the potential to be an ideal target for clinical intervention (Jeck et al., 2013). Currently, emerging evidence has demonstrated that they have been involved in multiple types of cancer and have great potentiality in regulating malignant tumor occurrence and development. For example, hsa_circ_0061825 could promote breast cancer progression, circ_SMAD4 contributes to gastric carcinogenesis, and circRNA-002178 acts as the tumor promoter in lung adenocarcinoma (Pan et al., 2020; Wang J. et al., 2020; Wang et al., 2021). Moreover, studies have found that different circRNAs have been shown to exhibit different effects and may act as the cancer-promoting or anticancer role in cancers. Such as in hepatocellular carcinoma, circTRIM33-12 could inhibit the progression, and circ_0008305 contributes to tumorigenesis (Zhang P. F. et al., 2019; Yan et al., 2021). However, the role of circRNAs in OS remains unclear. In this paper, we analyzed the most upregulated circRNAs in OS cell lines from GSE96964 and focused on the role and underlying mechanism of hsa_circ_0000073.

Based on our results, we verified hsa_circ_0000073 was highly expressed in OS cells and acted as an essential promotion factor in OS cells. Previously, Li X. et al. (2020) reported that hsa_circ_0000073 could enhance the proliferative, migrative, and invasive abilities of OS cells, which is also observed in our work. However, for the first time, our study revealed that the S phase of OS cells could arrest by hsa_circ_0000073 silencing. To explore the complex underlying mechanisms, we used the Affymetrix Gene Chip to analyze the DEGs after transfected sh-hsa_circ_0000073 in MG-63 and Saos-2 cells. Considering bioinformatics approaches are critical for handling and analyzing the deluge of information, bioinformatics analysis was performed to improve the accuracy of predicting target genes in our study. KEGG pathways analysis revealed that 16 DEGs were co-downregulated by hsa_circ_0000073 silencing in MG-63 and Saos-2 cell lines in “pathways in cancer.” It is widely accepted that circRNAs are important in ceRNA networks. In OS, previous studies have reported that circ_0000337, hsa_circ_0136666, and hsa_circ_0032463 act as ceRNA contribute to OS progression (Fang and Long, 2020; Zhang et al., 2020; Qin and Wu, 2021). In our study, we searched the databases and found four miRNAs with several potential binding sites of hsa_circ_0000073 and created a potential ceRNA network among hsa_circ_0000073, 4 miRNAs, and the 16 target genes identified earlier.

Next, we deeply investigate the miRNAs with binding sites of hsa_circ_0000073, which found miR-1252-5p was regulated by hsa_circ_0000073 and was lowly in OS cells. Emerging researches have shown that miRNAs could function as a carcinogen or tumor suppressor involved in cancer progression (Song et al., 2019; Zhang L. et al., 2019). Indeed, miR-1252-5p has been verified to suppress cell proliferation and migration in several cancers (Gu and Liu, 2020; Chen et al., 2021). However, the role of miR-1252-5p in OS is unclear. In this paper, we first observed miR-1252-5p could suppress the malignant behavior of OS cells.

Subsequently, after knockdown of hsa_circ_0000073, the marked downregulation in messenger RNA and protein expression of two cancer-related genes, CCNE2 and MDM2, was verified by qRT-PCR and WB. Moreover, our data also demonstrated that CCNE2 and MDM2 have a higher expression level in MG-63 and Saos-2 cells than hFOB 1.19 cells. As previously reported, CCNE2, which is frequently observed in proliferation, or migration in various kinds of cancers, is one of the two regulatory subunits of cyclin-dependent kinase 2 and could regulate the progression from G1 to S phase in cells (Sonntag et al., 2018; Sun et al., 2020; Wu D. et al., 2020). On the other hand, several studies have confirmed that MDM2 has oncogenic properties, and targeted MDM2 is an especially attractive therapeutic strategy (Egorova et al., 2020; Konopleva et al., 2020). Here, we also observed CCNE2 and MDM2 could enhance the development of OS and could be regulated by hsa_circ_0000073. It suggested that hsa_circ_0000073 may prompt the progression of OS by sponging miR-1252-5p and modulating CCNE2 and MDM2 simultaneously.

However, although we have several preliminary findings to indicate the significance of hsa_circ_0000073/miR-1252-5p/CCNE2 or MDM2 axis in regulating OS progression, these conclusions were based on the responses of cell lines and mice. In the future, we believe that the clinical relevance and more animal experiments such as the survival curve and reverse experiment should be enrolled in our study. Also, more molecules and pathways working with hsa_circ_0000073 needed to be deeply investigated.

## Conclusion

Our study confirms the key role of hsa_circ_0000073 in the regulation of malignant tumor behavior in OS cells, including proliferation, migration, invasion, and cell cycle *in vitro* as well as tumorigenesis *in vivo*. On the mechanisms, we first uncover hsa_circ_0000073 functions as a miR-1252-5p sponge and then affect CCNE2 and MDM2 expression, which providing a potential target for clinical treatment or prognosis of OS in the future.

## Data Availability Statement

The datasets presented in this study can be found in online repositories. The names of the repository/repositories and accession number(s) can be found below: GEO database and assigned GEO accession numbers as GSE176373.

## Ethics Statement

This work was approved by the Animal Ethics Committee of the Guizhou Provincial People’s Hospital.

## Author Contributions

XT, ZY, and ZR conceived and designed the experiments. ZR, QY, and JG performed the experiments. BL and ZY performed the statistical analysis. ZR and HH wrote the manuscript. All authors read and approved the final manuscript.

## Conflict of Interest

The authors declare that the research was conducted in the absence of any commercial or financial relationships that could be construed as a potential conflict of interest.

## Publisher’s Note

All claims expressed in this article are solely those of the authors and do not necessarily represent those of their affiliated organizations, or those of the publisher, the editors and the reviewers. Any product that may be evaluated in this article, or claim that may be made by its manufacturer, is not guaranteed or endorsed by the publisher.

## Supplementary Material

Supplementary Figure 1 qRT-PCR analyzed the efficiency of miR-1252-5p mimics and sponge.

Supplementary Figure 2 qRT-PCR and WB detected the knockdown effects of sh-CCNE2 and sh-MDM2.
