# Supplementary figures and images for: Circular RNA hsa_circ_0000073 Enhances Osteosarcoma Cells Malignant Behavior by Sponging miR-1252-5p and Modulating CCNE2 and MDM2
Source: Front Cell Dev Biol. 2021 Sep 9;9:714601. doi: 10.3389/fcell.2021.714601 (PMC8459753; doi:10.3389/fcell.2021.714601)

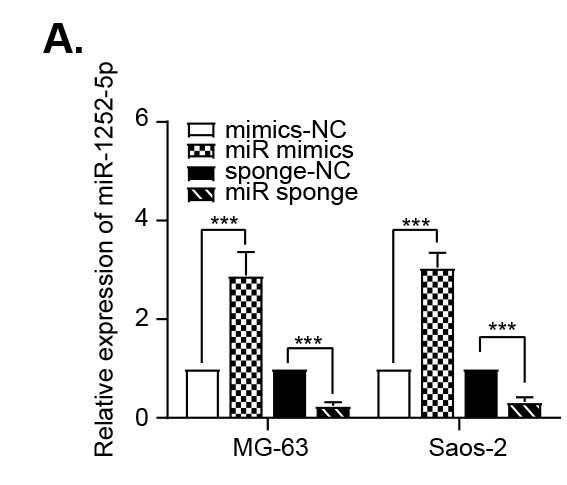

Supplement: Supplementary Figure 1 — qRT-PCR analyzed the efficiency of miR-1252-5p mimics and sponge. [file Image_1.TIF]

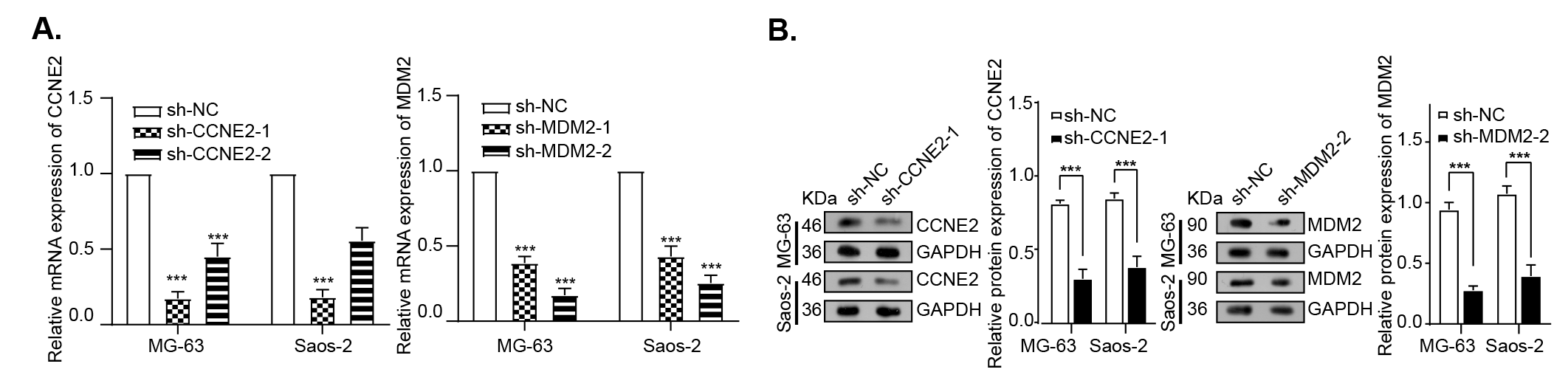

Supplement: Supplementary Figure 2 — qRT-PCR and WB detected the knockdown effects of sh-CCNE2 and sh-MDM2. [file Image_2.TIF]
