# Supplementary material for: Circular RNA hsa_circ_0000073 Enhances Osteosarcoma Cells Malignant Behavior by Sponging miR-1252-5p and Modulating CCNE2 and MDM2
Source: Front Cell Dev Biol. 2021 Sep 9;9:714601. doi: 10.3389/fcell.2021.714601 (PMC8459753; doi:10.3389/fcell.2021.714601)
Supplement: Supplementary file 3 [file Table_1.DOCX]

**Table S1. Sequences of shRNAs**

| shRNAs | Sequence (5'->3') |
| --- | --- |
| sh-hsa_circ_0000073-1 | GCUGCAAAGGGGCAUAAGGAATT |
| sh-hsa_circ_0000073-2 | [GCUUGCUGCAAAGGGGCAUAATT](http://blast.ncbi.nlm.nih.gov/Blast.cgi?PROGRAM=blastn&PAGE_TYPE=BlastSearch&LINK_LOC=blasthome&QUERY=%3ehsa_circ_0000073-siRNA2%0AGCTTGCTGCAAAGGGGCATAA&DATABASE=nr&EQ_MENU=Homo%C2%A0sapiens%C2%A0(taxid:9606)) |
| sh-CCNE2-1 | GGAACAAGUGAUUUCUCCAGAUUUA |
| sh-CCNE2-2 | AGGAGAGCAGAUAUGUUCAUGACAA |
| sh-MDM2-1 | GAGAUUUGUUUGGCGUGCCAAGCUU |
| sh-MDM2-2 | GAGUUUGUUGGGCGUCCAAGUACUU |
| sh-NC | UUCUCCGAACGUGUCACGUTT |
