# Supplementary material for: Circular RNA hsa_circ_0000073 Enhances Osteosarcoma Cells Malignant Behavior by Sponging miR-1252-5p and Modulating CCNE2 and MDM2
Source: Front Cell Dev Biol. 2021 Sep 9;9:714601. doi: 10.3389/fcell.2021.714601 (PMC8459753; doi:10.3389/fcell.2021.714601)
Supplement: Supplementary file 4 [file Table_2.DOCX]

**Table S2. Primer sequences for qRT-PCR**

| Primers | Sequence (5'->3') |
| --- | --- |
| hsa_circ_0000073 | Forward TGTGCCAGTGGATACAGTCT |
|  | Reverse TCCTTATGCCCCTTTGCAGC |
| CCNE2 | Forward TCAAGACGAAGTAGCCGTTTAC |
|  | Reverse TGACATCCTGGGTAGTTTTCCTC |
| MDM2 | Forward GGTGCTGTAACCACCTCACA |
|  | Reverse TGGCACGCCAAACAAATCTC |
| GAPDH | Forward ATGACATCAAGAAGGTGGTGAAGCAGG |
|  | Reverse GCGTCAAAGGTGGAGGAGTGGGT |
| miR-1252-5p | Forward GTCGTATCCAGTGCAGGGTCCGAGGTGCACTGGATACGACTAAATGA |
|  | Reverse TGCGGAGAAGGAAATTGAATTCA |
| U6 | Forward  GCTCGCTTCGGCAGCACA |
|  | Reverse  AACGCTTCACGAATTTGCGTG |
