# Supplementary material for: Circular RNA hsa_circ_0000073 Enhances Osteosarcoma Cells Malignant Behavior by Sponging miR-1252-5p and Modulating CCNE2 and MDM2
Source: Front Cell Dev Biol. 2021 Sep 9;9:714601. doi: 10.3389/fcell.2021.714601 (PMC8459753; doi:10.3389/fcell.2021.714601)
Supplement: Supplementary file 5 [file Table_3.DOCX]

**Table S3. Fold change of mRNA in different cells △**

| Probe set | Gene | Description | Fold change | |
| --- | --- | --- | --- | --- |
|  | Symbol |  | MG-63 | Saos-2 |
| TC1100013088.hg.1 | BIRC2 | baculoviral IAP repeat containing 2 | 3.031 | 3.095 |
| TC0800011148.hg.1 | CCNE2 | cyclin E2 | 3.758 | 3.506 |
| TC1200010977.hg.1 | CDK4 | cyclin-dependent kinase 4 | 1.58 | 1.659 |
| TC0100017730.hg.1 | EGLN1 | egl-9 family hypoxia-inducible factor 1 | 2.014 | 2.166 |
| TC0200007458.hg.1 | EPAS1 | endothelial PAS domain protein 1 | 1.625 | 2.114 |
| TC1200007882.hg.1 | GLI1 | GLI family zinc finger 1 | 1.778 | 1.682 |
| TC0900012160.hg.1 | GNG10 | guanine nucleotide binding protein (G protein), gamma 10 | 1.636 | 1.558 |
| TC0700008351.hg.1 | GNG11 | guanine nucleotide binding protein (G protein), gamma 11 | 2.189 | 1.636 |
| TC0300009916.hg.1 | HES1 | hes family bHLH transcription factor 1 | 1.613 | 2.514 |
| TC2100008494.hg.1 | IFNAR2 | interferon (alpha, beta and omega) receptor 2 | 2.014 | 2.099 |
| TC1400010796.hg.1 | JAG2 | jagged 2 | 1.548 | 1.729 |
| TC1200008116.hg.1 | MDM2 | MDM2 proto-oncogene, E3 ubiquitin protein ligase | 1.625 | 2.412 |
| TC0400008813.hg.1 | MGST2 | microsomal glutathione S-transferase 2 | 1.905 | 1.741 |
| TC1400009705.hg.1 | PGF | placental growth factor | 4.287 | 2.585 |
| TC0100008874.hg.1 | PRKACB | protein kinase, cAMP-dependent, catalytic, beta | 1.945 | 1.753 |
| TC0300006925.hg.1 | TGFBR2 | transforming growth factor beta receptor II | 1.853 | 1.853 |

△sh-hsa_circ_0000073 group VS sh-NC group
