# Supplementary material for: Circular RNA hsa_circ_0000073 Enhances Osteosarcoma Cells Malignant Behavior by Sponging miR-1252-5p and Modulating CCNE2 and MDM2
Source: Front Cell Dev Biol. 2021 Sep 9;9:714601. doi: 10.3389/fcell.2021.714601 (PMC8459753; doi:10.3389/fcell.2021.714601)
Supplement: Supplementary file 6 [file Table_4.DOCX]

**Table S4. Protein-protein interactions**

| Node1 | Node2 | Homology | Coexpression | Experimentally  Determined  Interaction | Database  Annotated | Automated  Textmining | **Combined**  **Score** |
| --- | --- | --- | --- | --- | --- | --- | --- |
| BIRC2 | CCND1 | 0 | 0 | 0 | 0 | 0.566 | 0.566 |
| BIRC2 | MDM2 | 0 | 0.063 | 0 | 0 | 0.411 | 0.425 |
| BIRC2 | TP53 | 0 | 0 | 0 | 0 | 0.586 | 0.586 |
| CCNA2 | CCND1 | 0.694 | 0.084 | 0.065 | 0.72 | 0.885 | 0.809 |
| CCNA2 | CDK4 | 0 | 0.184 | 0.994 | 0.9 | 0.857 | 0.999 |
| CCNA2 | MDM2 | 0 | 0.062 | 0.108 | 0.9 | 0.58 | 0.96 |
| CCNA2 | CCND2 | 0.685 | 0.062 | 0.065 | 0.9 | 0.772 | 0.927 |
| CCNA2 | CCNE1 | 0.645 | 0.393 | 0.168 | 0.9 | 0.773 | 0.96 |
| CCNA2 | CDK2 | 0 | 0.403 | 0.998 | 0.9 | 0.973 | 0.999 |
| CCNA2 | RB1 | 0 | 0.108 | 0.451 | 0.9 | 0.698 | 0.983 |
| CCNA2 | TP53 | 0 | 0.091 | 0.354 | 0.9 | 0.857 | 0.99 |
| CCNA2 | CCNE2 | 0.64 | 0.431 | 0.168 | 0.9 | 0.771 | 0.962 |
| CCND1 | JAG2 | 0 | 0.065 | 0 | 0 | 0.416 | 0.43 |
| CCND1 | TGFBR2 | 0 | 0.061 | 0 | 0 | 0.427 | 0.438 |
| CCND1 | CCNE2 | 0.671 | 0.063 | 0.057 | 0.36 | 0.752 | 0.535 |
| CCND1 | MDM2 | 0 | 0 | 0.379 | 0 | 0.725 | 0.821 |
| CCND1 | CCND2 | 0.961 | 0.061 | 0 | 0.9 | 0.827 | 0.905 |
| CCND1 | GLI1 | 0 | 0 | 0.05 | 0.8 | 0.649 | 0.927 |
| CCND1 | CCNE1 | 0.67 | 0.063 | 0.057 | 0.9 | 0.844 | 0.93 |
| CCND1 | TP53 | 0 | 0.061 | 0.379 | 0 | 0.942 | 0.963 |
| CCND1 | RB1 | 0 | 0.062 | 0.522 | 0.9 | 0.858 | 0.992 |
| CCND1 | CDK2 | 0 | 0.089 | 0.845 | 0.9 | 0.96 | 0.999 |
| CCND1 | CDK4 | 0 | 0.082 | 0.994 | 0.9 | 0.973 | 0.999 |
| CCND2 | GLI1 | 0 | 0 | 0.05 | 0.8 | 0.519 | 0.9 |
| CCND2 | CDK4 | 0 | 0.064 | 0.918 | 0.9 | 0.933 | 0.999 |
| CCND2 | MDM2 | 0 | 0 | 0 | 0 | 0.473 | 0.473 |
| CCND2 | CCNE2 | 0.664 | 0.064 | 0.057 | 0.36 | 0.777 | 0.544 |
| CCND2 | TP53 | 0 | 0 | 0 | 0 | 0.644 | 0.644 |
| CCND2 | CCNE1 | 0.645 | 0.064 | 0.18 | 0.9 | 0.77 | 0.939 |
| CCND2 | RB1 | 0 | 0.062 | 0.522 | 0.9 | 0.742 | 0.986 |
| CCND2 | CDK2 | 0 | 0.063 | 0.776 | 0.9 | 0.794 | 0.995 |
| CCNE1 | GLI1 | 0 | 0 | 0.085 | 0 | 0.391 | 0.419 |
| CCNE1 | CDK4 | 0 | 0.143 | 0.994 | 0.9 | 0.824 | 0.999 |
| CCNE1 | MDM2 | 0 | 0.062 | 0.108 | 0 | 0.558 | 0.597 |
| CCNE1 | CCNE2 | 0.928 | 0.124 | 0 | 0.9 | 0.743 | 0.913 |
| CCNE1 | TP53 | 0 | 0.063 | 0.123 | 0.9 | 0.771 | 0.978 |
| CCNE1 | RB1 | 0 | 0.064 | 0.576 | 0.9 | 0.603 | 0.982 |
| CCNE1 | CDK2 | 0 | 0.162 | 0.999 | 0.9 | 0.928 | 0.999 |
| CCNE2 | CDK4 | 0 | 0.111 | 0.265 | 0.8 | 0.77 | 0.965 |
| CCNE2 | MDM2 | 0 | 0.062 | 0.108 | 0 | 0.366 | 0.423 |
| CCNE2 | CDK2 | 0 | 0.199 | 0.997 | 0.9 | 0.856 | 0.999 |
| CCNE2 | RB1 | 0 | 0.064 | 0.372 | 0.9 | 0.414 | 0.96 |
| CCNE2 | TP53 | 0 | 0.063 | 0.304 | 0.9 | 0.612 | 0.971 |
| CDK2 | CDK4 | 0.911 | 0.119 | 0.126 | 0.9 | 0.952 | 0.926 |
| CDK2 | MDM2 | 0 | 0.05 | 0 | 0.9 | 0.697 | 0.968 |
| CDK2 | TP53 | 0 | 0.094 | 0.716 | 0.9 | 0.924 | 0.997 |
| CDK2 | RB1 | 0 | 0.081 | 0.994 | 0.9 | 0.709 | 0.999 |
| CDK4 | GLI1 | 0 | 0.055 | 0.154 | 0 | 0.417 | 0.493 |
| CDK4 | MDM2 | 0 | 0 | 0 | 0 | 0.798 | 0.798 |
| CDK4 | TP53 | 0 | 0.093 | 0.186 | 0 | 0.843 | 0.874 |
| CDK4 | RB1 | 0 | 0.063 | 0.994 | 0.9 | 0.861 | 0.999 |
| EGLN1 | EPAS1 | 0 | 0.065 | 0.806 | 0.9 | 0.866 | 0.997 |
| FANCE | HES1 | 0 | 0 | 0.993 | 0.9 | 0.1 | 0.999 |
| GLI1 | MDM2 | 0 | 0 | 0.053 | 0 | 0.426 | 0.433 |
| GLI1 | JAG2 | 0 | 0.062 | 0.05 | 0 | 0.44 | 0.458 |
| GLI1 | TP53 | 0 | 0 | 0.112 | 0 | 0.567 | 0.599 |
| GLI1 | PRKACB | 0 | 0.078 | 0.166 | 0.9 | 0.137 | 0.924 |
| GNB3 | PRKACB | 0 | 0.209 | 0 | 0.6 | 0.157 | 0.709 |
| GNB3 | GNB4 | 0.981 | 0 | 0 | 0.9 | 0.756 | 0.902 |
| GNB3 | GNG11 | 0 | 0.061 | 0.996 | 0.9 | 0.379 | 0.999 |
| GNB3 | GNG10 | 0 | 0.061 | 0.994 | 0.9 | 0.332 | 0.999 |
| GNB4 | PRKACB | 0 | 0.209 | 0 | 0.6 | 0.157 | 0.709 |
| GNB4 | GNG11 | 0 | 0.083 | 0.997 | 0.9 | 0.598 | 0.999 |
| GNB4 | GNG10 | 0 | 0.061 | 0.994 | 0.9 | 0.488 | 0.999 |
| GNG10 | GNG11 | 0 | 0 | 0 | 0.9 | 0.908 | 0.99 |
| GNG10 | PRKACB | 0 | 0 | 0.08 | 0.6 | 0 | 0.616 |
| GNG11 | PRKACB | 0 | 0 | 0.08 | 0.6 | 0.117 | 0.646 |
| HES1 | JAG2 | 0 | 0 | 0.132 | 0 | 0.494 | 0.542 |
| HES1 | RB1 | 0 | 0 | 0 | 0.9 | 0.074 | 0.903 |
| JAG2 | TP53 | 0 | 0 | 0 | 0 | 0.4 | 0.4 |
| MDM2 | RB1 | 0 | 0.061 | 0.379 | 0.9 | 0.57 | 0.971 |
| MDM2 | TP53 | 0 | 0.236 | 0.987 | 0.9 | 0.974 | 0.999 |
| RB1 | TP53 | 0 | 0.061 | 0.379 | 0.9 | 0.789 | 0.986 |
| TGFBR2 | TP53 | 0 | 0 | 0.05 | 0 | 0.559 | 0.563 |
